# Editorial: Non-pharmacological interventions for mental disorders

**DOI:** 10.3389/fpsyg.2024.1363348

**Published:** 2024-03-22

**Authors:** Lara Guedes de Pinho, César Fonseca, Łukasz Gawęda, Manuel Lopes, Brooke C. Schneider

**Affiliations:** ^1^Nursing Department, University of Évora, Évora, Portugal; ^2^Comprehensive Health Research Centre (CHRC), University of Évora, Évora, Portugal; ^3^Experimental Psychopathology Lab, Institute of Psychology, Polish Academy of Sciences, Warsaw, Poland; ^4^Department of Psychiatry and Psychotherapy, University Medical Center Hamburg-Eppendorf, Hamburg, Germany

**Keywords:** mental health, depression, psychosis, lifespan perspective, scalability, wellbeing, psychotherapy

International evidence-based treatment guidelines recommend non-pharmacological interventions as gold standard approaches for the treatment of most mental disorders. Despite this, a large treatment gap exists such that many individuals suffering from mental disorders receive only medication or no treatment at all (Kazdin, 2017). The World Health Organization has declared improving mental health a top priority for worldwide sustainability (World Health Organization, 2019) and recommended several transformations of world mental health practices to improve access for all individuals (World Health Organization, 2022). Inspired by this, in this Research Topic, we sought to support dissemination of research on all types of non-pharmacological interventions for mental health and thus, highlight possible avenues for closing the mental health treatment gap and improving mental health care.

Promotion of cost-effective preventative approaches is necessary to promote good mental health globally and to prevent the onset of mental disorders (World Health Organization, 2022). The systematic umbrella review by Saijonkari et al. aimed to evaluate the effectiveness of interventions for the promotion of mental health and mental wellbeing, as well as for the primary prevention of mental health disorders. This review found evidence primarily for interventions that utilize cognitive-behavioral therapy (CBT) and promote resilience, mindfulness or healthy lifestyles. Interventions such as motivational interviewing to reduce alcohol consumption in young adults, parenting interventions and workplace interventions are also covered.

Primary treatment approaches for anxiety include psychotherapy as well as pharmacotherapy; however, the use of alternative treatment approaches may help reach more individuals suffering from symptoms, especially treatments which can be administered by professionals without specialized training in mental healthcare. The meta-analysis by Hong et al. evaluated the efficacy of electroacupuncture (EA) for patients with anxiety, concluding that this intervention significantly reduced patients' anxious symptomatology, suggesting it as an effective therapeutic option.

Development of sustainable long-term care models for treatment of severe mental illness is necessary to reduce healthcare costs and support symptom stability. Yet, little is known about patients' perceptions of these programs. Rohenkohl et al. examined patients' preferences in relation to integrated care provided in the “Hamburg Model (ACCESS),” a multimodal integrated care concept in which treatment is adapted to an individual patient's needs by a therapeutically oriented community treatment team. Having an assigned long-term therapist with whom the patient developed a trusting therapeutic relationship and having 24/7 telephone contact for crises were factors considered by patients to be the most useful and important.

Empirically-based concepts for psychological care during acute inpatient stays for individuals with psychosis are lacking (American Psychiatric Association, 2021). Fischer et al. report a pilot study on the feasibility, acceptability and safety of Metacognitive Training for patients with psychosis in a psychiatric acute care setting (MCT-Acute). Although MCT-Acute represents a promising non-pharmacological approach for improving treatment of acute psychosis, controlled trials are needed to confirm its efficacy.

The WHO Mental Health report calls for increased dissemination of community-based programs (2022). Machorrinho et al. present data on an 8-week psychomotor therapy program for victims of intimate partner violence. The Feel-Own-Move (FOM) intervention was administered in shelter homes to victims of intimate partner violence (IPV). The authors conclude that FOM appears to be a viable psychomotor therapy intervention for female victims of IPV living in shelters and leads to reduced body dissociation among participants, which is suggested to prospectively contribute to improved mental health and quality of life.

Modulation of dysfunctional brain activation via transcranial electrical stimulation is emerging as a possible new treatment for attention-deficit hyperactivity disorder (ADHD). In their study Kannen et al. aimed to reduce symptoms and improve attention in adults with ADHD by enhancing alpha band power via transcranial alternating current stimulation (tACS). Participants received active and sham stimulation on distinct days. The authors concluded that the study did not provide clear evidence of an increase in alpha power induced by tACS, so observed improvements in attention could not be attributed to intervention-related effects. Despite this, the authors discuss limitations to their work and provide suggestions for improving future studies exploring whether alpha power enhancement via tACS could be a therapeutic option for ADHD.

Higher education students are a group at risk of developing mental health problems, and these problems worsened with the COVID-19 pandemic, with college students reporting increased rates of depression and anxiety (Li et al., 2021). In this Research Topic, Zuo and Zhang report an RCT on a CBT-based intervention for university students with maladaptive perfectionism. In line with the authors' hypothesis, the intervention led to improvements in maladaptive aspects of perfectionism, concern about mistakes and doubts about actions, as well as symptoms of anxiety and depression compared to a wait-list control group.

Expanding research to improve understanding of the effectiveness of empirically based treatments outside of strictly controlled trials is essential for transforming mental healthcare. Wallsten et al. assumed a transdiagnostic approach to examining the effects of a group rumination-focused CBT (RF-CBT) vs. a wait-list control in a primary care setting utilizing a sample of patients with depression, anxiety and/or insomnia as well as other comorbidities. RF-CBT led to significant improvements in insomnia, whereas improvement in depression was detected only at 2-month follow-up and no significant group differences were found for rumination. It remains unclear which mechanisms may have contributed to improvements in insomnia and depression, but this study suggests a need for further examination of the impacts of RF-CBT on insomnia.

Schneider et al. report mediators of depression reduction in Metacognitive Training for depression in older adults (MCT-Silver), a CBT-based group intervention. In line with WHO recommendations for mental health transformations, due to its structured format, MCT-Silver can be administered by individuals without advanced training in mental health and is easily scalable (www.uke.de/mct-silver). In a recently published RCT, MCT-Silver led to significant reductions in depression and rumination compared to an active control group (Schneider et al., 2024).

Language and cultural adaptations of empirically-based psychological interventions are necessary to increase dissemination and promote worldwide mental health care. Pinho et al. report details of a planned RCT on a translated and culturally-adapted version of MCT-Silver in Portugal. Promotion of mental healthcare treatment for late life depression is especially important as depression in older adults often goes undetected and is undertreated (Horackova et al., 2019).

The WHO's Sustainable Development Goals (SDGs) set reduction of suicide as an international priority by aiming to reduce the suicide mortality rate by a third by 2030 (World Health Organization, 2022). Particularly frontline medical works faced enormous psychological stress during the COVID-19 pandemic and represent a vulnerable group (Ghebreyesus, 2020). In an uncontrolled pilot trial, Robles et al. report the implementation and effects of a brief, remote manualized crisis intervention and suicide risk management intervention for COVID-19 healthcare workers in Mexico. In their study, frontline workers were invited to contact a free 24-h helpline. Trained psychologists and psychiatrists provided up to 12 crisis intervention sessions either online or by telephone utilizing empirically based techniques (EBTs). Helpline users demonstrated significant improvements in Clinical Global Impression (CGI) severity score and self-rated distress. This intervention provides initial evidence of the feasibility of a low-threshold intervention for crisis management.

In a final study on the treatment of affective disorders, in an uncontrolled pilot study, Oaks-Cornellissen et al. examined effects of a 10-week multimodal lifestyle intervention for improving the mental health of individuals with an affective disorder in South Africa. Differing from other approaches, the program focused on promotion of healthy lifestyle behaviors as well as positive psychology practices. Significant improvements in many domains, including overall mental health, depression, anxiety and vitality are reported. This work provides evidence of the feasibility and acceptance of the intervention in patients with clinical diagnoses.

Finally, reflecting the need for improved treatments across the lifespan (World Health Organization, 2022), three studies examined interventions in children and/or their families. Reduction of repetitive and off task behaviors is a major goal in the treatment of many children with autism spectrum disorder (ASD); however, it remains unclear to what extent some sensory integration therapies, and specifically compression (Trembath et al., 2023), may lead to reduction of problematic behaviors. In a study by Grandits et al., nine children with ASD were randomly assigned to wear compression clothing for either their first five or last five sessions of Applied Behavioral Analysis. Compression clothes failed to increase task participation or reduce repetitive behaviors so that it could not be identified as an effective add-on approach to treatment of ASD.

Children with learning disorders (LDs) have higher rates of emotional disorders and may be at a greater risk for poorer self-concepts compared to typically developing peers (Huang, 2011). Martínez-Briones et al. examined the effect of neurofeedback (NFB) on self-concept among 34 children ages 8–11 years with a learning disorder vs. a sham-NFB or a waiting-list control group. Supporting the author's hypotheses, NFB led to significant increases in several domains of self-concept suggesting that NFB may represent a promising new path to improving treatment for children with LDs.

Targeting parenting practices and strengthening resources for families are key areas of mental health transformations (World Health Organization, 2022). In a final study for this Research Topic, Marlotte et al. adapted a trauma-informed, resilience skill-building family intervention for adolescents ages 12–18 years old with depression and a participating caregiver. Twenty-five pairs were randomized to either the adapted intervention [Families Over Coming Under Stress for Families with Adolescent Depression (FOCUS-AD)] or usual care (CBT) delivered in school-based health clinics. FOCUS-AD was found to be feasible and acceptable; however, depressive symptoms declined significantly in both groups. Contrary to expectations, family functioning was not significantly improved in either intervention. Although this study contributes to research indicating that skills-based interventions may improve depressive symptoms particularly in minority and under-resourced youth and families, it remains unclear how important outcomes such as family functioning may be better targeted.

## Author contributions

LP: Writing—review & editing. CF: Writing—review & editing. ŁG: Writing—review & editing. ML: Writing—review & editing. BS: Writing—review & editing, Writing—original draft.
